# Ribosome engineering to promote new crystal forms

**DOI:** 10.1107/S0907444912006348

**Published:** 2012-04-17

**Authors:** Maria Selmer, Yong-Gui Gao, Albert Weixlbaumer, V. Ramakrishnan

**Affiliations:** a Department of Cell and Molecular Biology, Uppsala University, Box 596, SE-751 24 Uppsala, Sweden; b MRC Laboratory of Molecular Biology, Hills Road, Cambridge CB2 0QH, England

**Keywords:** ribosome, GTPase, engineering

## Abstract

Crystallographic studies of the ribosome have provided molecular details of protein synthesis. However, the crystallization of functional complexes of ribosomes with GTPase translation factors proved to be elusive for a decade after the first ribosome structures were determined. Analysis of the packing in different 70S ribosome crystal forms revealed that regardless of the species or space group, a contact between ribosomal protein L9 from the large subunit and 16S rRNA in the shoulder of a neighbouring small subunit in the crystal lattice competes with the binding of GTPase elongation factors to this region of 16S rRNA. To prevent the formation of this preferred crystal contact, a mutant strain of *Thermus thermophilus*, HB8-MRCMSAW1, in which the ribosomal protein L9 gene has been truncated was constructed by homologous recombination. Mutant 70S ribosomes were used to crystallize and solve the structure of the ribosome with EF-­G, GDP and fusidic acid in a previously unobserved crystal form. Subsequent work has shown the usefulness of this strain for crystallization of the ribosome with other GTPase factors.

## Abstract 2

Truncation of ribosomal protein L9 in *T. thermophilus* allows the generation of new crystal forms and the crystallization of ribosome–GTPase complexes.

## 1. Introduction

The ribosome is responsible for the translation of mRNA into protein. Structural understanding of this process was greatly advanced by the determination of the complete structures of the 50S ribosomal subunit from *Haloarcula marismortui* (Ban *et al.*, 2000 ▶) and the 30S ribosomal subunit from *Thermus thermophilus* (Wimberly *et al.*, 2000 ▶). Subsequently, these structures were used to interpret lower resolution structures of the entire 70S ribosome in complex with tRNA ligands (Yusupov *et al.*, 2001 ▶) as well as with release factors (Petry *et al.*, 2005 ▶) and to phase higher resolution structures of the ribosome such as the empty *Escherichia coli* ribosome (Schuwirth *et al.*, 2005 ▶) and the 70S ribosome in complex with tRNA and mRNA (Selmer *et al.*, 2006 ▶).

During the translation cycle, GTPase translational factors interact with the ribosome at each of the major stages of initiation (IF2), elongation (EF-Tu and EF-G), termination (RF3) and recycling (EF-G). These factors have all been visualized on the ribosome by cryo-electron microscopy (cryo-EM; Connell *et al.*, 2007 ▶; Gao *et al.*, 2007 ▶; Schuette *et al.*, 2009 ▶; Myasnikov *et al.*, 2005 ▶; Villa *et al.*, 2009 ▶). Despite the increasing resolution of cryo-EM structures, which is currently at about 6–7 Å (Schuette *et al.*, 2009 ▶; Villa *et al.*, 2009 ▶), X-ray crystallography is still the only technique that has been able to directly observe detailed interactions in ribosomal complexes (see, for example, Ogle *et al.*, 2001 ▶; Selmer *et al.*, 2006 ▶; Hansen *et al.*, 2002 ▶). Visualization of the details of these interactions is necessary in order to understand the action of GTPase factors, including the mechanism of GTP hydrolysis and how it is specifically triggered by the ribosome on each factor at the appropriate stage. However, for a decade after the determination of the first structures of ribosomal subunits, the crystallization of ribosomal complexes with translational GTPases proved to be elusive.

Here, we present a crystal-packing analysis of previous 70S crystal forms, revealing how preferred crystal contacts involving ribosomal protein L9 compete with the binding of GTPase factors to the ribosome, preventing the crystallization of ribosomal GTPase factor complexes. Furthermore, we describe the rational design of a mutant *T. thermophilus* strain HB8-MRCMSAW1 that cannot form the preferred crystal contact. Finally, we describe how mutant ribosomes from this strain were used to crystallize a 70S ribosome complex with EF-G and the antibiotic fusidic acid. This new crystal form allowed structure determination of the EF-G complex (Gao *et al.*, 2009 ▶). These mutant ribosomes were also used to crystallize the ribosome with EF-Tu during decoding (Schmeing *et al.*, 2009 ▶; Voorhees *et al.*, 2010 ▶) and with RF3 during termination (Jin *et al.*, 2011 ▶).

## 2. Materials and methods

### 2.1. Bacterial strains and plasmids

*T. thermophilus* strain HB8 was used as the starting strain for construction of the L9 truncated mutant. A pUC18 clone of the heat-stable kanamycin resistance (*HTK*) gene (Hoseki *et al.*, 1999 ▶) was a kind gift from A. Dahlberg (Brown University). The transformation of *T. thermophilus* was performed as described previously (Koyama *et al.*, 1986 ▶).

### 2.2. Construction of *T. thermophilus* HB8-MRCMSAW1

**Figure 1:**
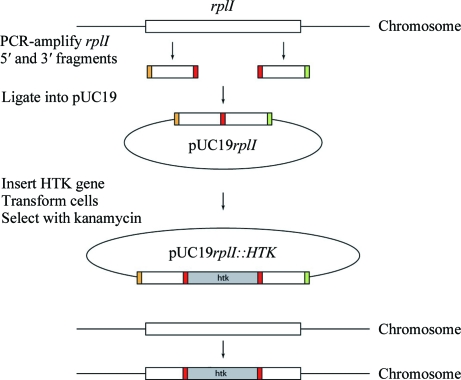
Experimental strategy for the production of *T. thermophilus* strain HB8-MRCMSAW1 using homologous recombination (Hashimoto *et al.*, 2001 ▶; Cameron *et al.*, 2004 ▶).

The L9 truncated mutant was prepared using homologous recombination with a pUC19 plasmid containing the *HTK* gene (Hashimoto *et al.*, 2001 ▶; Cameron *et al.*, 2004 ▶) flanked by sequences homologous to the region around the L9-encoding *rplI* gene of the *T. thermophilus* genome (GenBank accession No. AB103400) according to Fig. 1 ▶.

Two fragments of the *rplI* gene were PCR-amplified using the primers L9f1 (CAA**GGTACC**GCTTTCCGCCAAGGAGCAGAGGATC; *Kpn*I site shown in bold) and L9b1 (AAA­A**CTGCAG** CTAGGCCTGGGCGCGGATCCGG; *Pst*I site shown in bold, stop codon underlined), and L9f2 (AAAA**CTGCAG**CACCATTGACCCCAAGCGCCTGGC; *Pst*I site shown in bold) and L9b2 (GGT**AAGCTT**CCCCTTGGCCGTGAGCAACCGG; *Hin*dIII site shown in bold), and ligated into pUC19 cleaved using *Kpn*I and *Hin*dIII. The resulting plasmid was cleaved using *Pst*I and the *HTK* gene amplified with the primers Htkf (CCA**CTGCAG**GGTACCCGTTGACGGCGGATATGG; *Pst*I site shown in bold) and Htkb (GGT**CTGCAG**CGTAACCAACATGATTAACAATTATTAGAGG; *Pst*I site shown in bold) was inserted into the *Pst*I site. The resulting plasmid pL9_55_htk was transformed into *T. thermophilus* HB8 cells (Koyama *et al.*, 1986 ▶), which were grown on kanamycin-containing 162 plates to select for recombinants. Incorporation of the truncated L9 gene and the *HTK* gene by homologous recombination was confirmed by PCR and sequencing.

### 2.3. Preparation of EF-G, 70S and mRNA

*T. thermophilus* EF-G was cloned into vector pET42b to create a construct with a C-terminal His tag containing a TEV site and overexpressed in *E. coli* strain BL21 (DE3). The cells were lysed in lysis buffer (50 m*M* sodium phosphate pH 8.0, 0.3 *M* NaCl, 5 m*M* β-mercaptoethanol) using an Emulsiflex (Avestin, Ottawa, Canada) and the cell debris was pelleted by centrifugation for 30 min at 30 000*g*. The cell lysate was incubated at 338 K for 30 min and denatured *E. coli* proteins were pelleted by centrifugation. The supernatant was loaded onto an Ni–NTA agarose column (Qiagen) pre-equilibrated in lysis buffer. After washing with lysis buffer containing 20 m*M* imidazole, EF-G was eluted, dialyzed against TEV buffer (20 m*M* sodium phosphate pH 8.0, 0.3 *M* NaCl) and treated with TEV protease overnight. After the addition of 20 m*M* imidazole, the untagged EF-G was passed through an Ni–NTA agarose column (Qiagen). EF-G fractions were pooled and applied onto a HiLoad 26/60 Superdex 200 prep-grade column (Amersham Biosciences) in gel-filtration buffer (20 m*M* sodium phosphate pH 8.0, 0.3 *M* NaCl). The EF-G peak was dialyzed against ion-exchange buffer (50 m*M* Tris–HCl pH 7.0, 10 m*M* magnesium acetate) and subsequently loaded onto a HiPrep QXL column (GE Healthcare) equilibrated with the same buffer. EF-G was eluted with a linear gradient of 0–0.7 *M* NaCl in ten column volumes. Finally, EF-G was dialyzed against buffer *G* (5 m*M* HEPES–KOH pH 7.5, 50 m*M* KCl, 10 m*M* ammonium chloride, 10 m*M* magnesium acetate, 6 m*M* β-mercaptoethanol) and concentrated to 24.5 mg ml^−1^ using an Ultra concentrator (Amicon).

70S ribosomes from *T. thermophilus* HB8-MRCMSAW1 and *E. coli* tRNA^fMet^ were prepared using previously described methods (Selmer *et al.*, 2006 ▶). The mRNA Z4C was chemically synthesized (Dharmacon) with the sequence 5′-­GGCAAGGAGGUAAAA**AUGUUC**AAAA-3′, with an fMet codon at the P site (bold) and a Phe codon at the A site (underlined bold).

### 2.4. Complex formation, crystallization and structure determination

**Figure 2:**
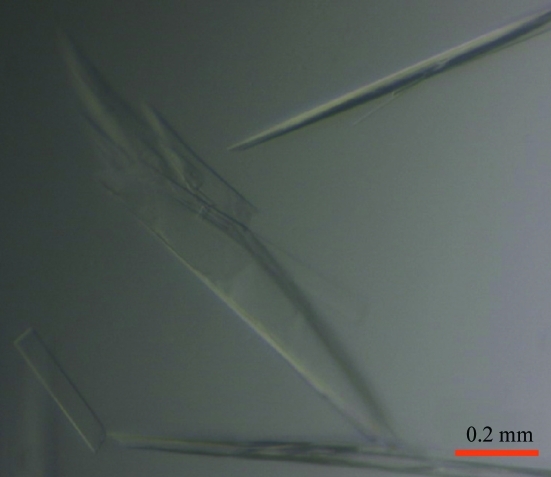
Optimized crystals of the 70S–EF-G complex with GDP and fusidic acid obtained using ΔL9 ribosomes.

70S ribosomes at final concentrations of 4.0 and 8.0 µ*M* mRNA were incubated in buffer *G* at 328 K for 6 min. 16.0 µ*M* tRNA^fMet^ was quickly added and the complex was incubated at 328 K for 6 min. At this point, 500 µ*M* fusidic acid, 20 µ*M* EF-G and 100 µ*M* GTP which had been pre-incubated at room temperature for 20 min were added to the ribosome complex and the resulting mixture was incubated for 20 min at 328 K and for 30 min at room temperature prior to crystallization. 2.3 µ*M* Deoxy Big CHAP (DOBC; Hampton Research) was added to the complex, giving a final concentration of 3.3 µ*M* 70S ribosomes. The complex was subjected to crystallization screening in sitting-drop vapour-diffusion experiments using 200 nl drops (Stock *et al.*, 2005 ▶). Initial small crystals grew in sitting drops using Hampton Research Crystal Screen and Crystal Screen 2 (0.1 *M* MES pH 6.5, 12% PEG 20K). After optimization to improve the crystal size and quality, 3 µl reservoir solution (0.1 *M* MES pH 6.5–6.6, 8.5–9.5% PEG 20K) was mixed with 3 µl complex solution and streak-seeded. Crystals grew in 5–14 d to dimensions of ∼20 × 100 × 500 µm (Fig. 2 ▶). Data collection, structure determination and refinement have been described elsewhere (Gao *et al.*, 2009 ▶).

## 3. Results and discussion

### 3.1. Analysis of crystal contacts in the factor-binding site of published 70S ribosomal crystal forms

In attempts to crystallize the relatively stable complex of the ribosome with EF-G in the presence of the antibiotic fusidic acid, we obtained diffracting crystals. When the structure was solved, to our disappointment no EF-G was visible. In place of EF-G, L9 of a neighbouring ribosome occupied the factor-binding site of the 30S subunit, suggesting that EF-G was competed off the ribosome during crystallization by the L9 crystal contact with the 30S shoulder (Selmer *et al.*, 2006 ▶). This observation led us to check the crystal contacts in other available 70S crystal forms.

**Figure 3:**
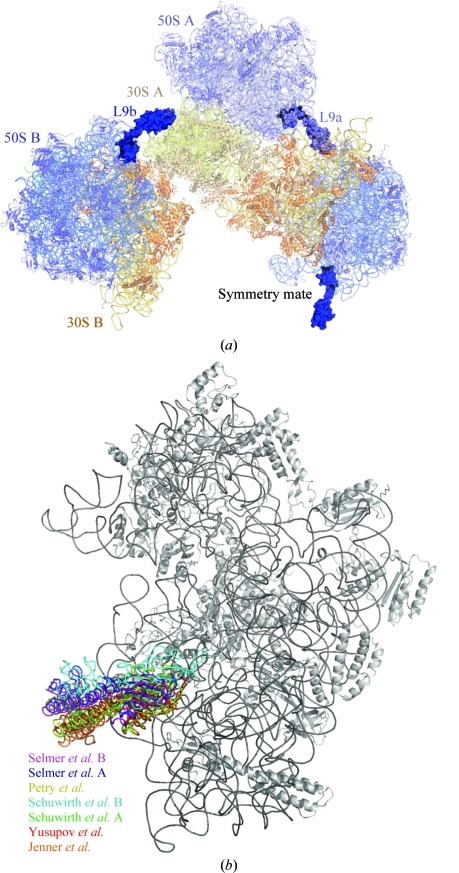
(*a*) Ribosomal protein L9 (dark blue) from one ribosome extends to engage its C-terminal domain in a crystal contact with 16S rRNA of another ribosome, as exemplified by the structure of *T. thermophilus* 70S in complex with mRNA and tRNA (Selmer *et al.*, 2006 ▶). 30S proteins and rRNA are shown in yellow. 50S proteins and rRNA are shown in blue. (*b*) Comparison of the L9 crystal contact with 16S rRNA in different 70S crystal forms. The structures were superimposed based on the shoulder of 16S rRNA. L9 is shown in different colours in the different structures.

Strikingly, all crystal forms of 70S ribosomes in published crystal structures (Petry *et al.*, 2005 ▶; Schuwirth *et al.*, 2005 ▶; Selmer *et al.*, 2006 ▶; Yusupov *et al.*, 2001 ▶) had crystal contacts between ribosomal protein L9 in the 50S subunit of one ribosome and the neighbouring 30S subunit of another ribosome. L9 consists of two globular domains linked by a long α-­helix (Hoffman *et al.*, 1994 ▶). In all of the different crystal forms, L9 extends from its binding site between 23S rRNA helices H15 and H76 below the L1 stalk to engage its C-­terminal domain (L9-C) in a crystal contact with the shoulder of the 30S subunit of a neighbouring ribosome (Fig. 3 ▶ *a*). L9 in a similar conformation extending from the ribosome has also been observed in single-particle cryo-EM studies of 70S complexes with EF-G and GTP (Spahn *et al.*, 2001 ▶).

Even though the crystal forms belong to different space groups and are from different species, *e.g. T. thermophilus* (Selmer *et al.*, 2006 ▶) or *E. coli* (Schuwirth *et al.*, 2005 ▶), the overall regions of contact between L9 and the neighbouring 30S subunit are similar. The 82–93 and 121–122 loop regions in L9-C contact the 56 and 357–360 regions of 16S rRNA helix 5 and the 368–369 region of 16S rRNA helix 15 using hydrogen bonds and stacking interactions (*E. coli* rRNA numbering). Despite the similarity, the exact inter­actions are not conserved: the hidden surface area varies between 140 and 460 Å^2^ and the relative position of the C-­terminal domain of L9 differs by up to 20 Å. In the three most extensive packing interactions, with more than 400 Å^2^ hidden surface area (Table 1 ▶), the position of L9-C differs by about 4 Å (Fig. 3 ▶ *b*).

The L9 contact occurs in the area of the 30S subunit where domains II of IF-2 (Myasnikov *et al.*, 2005 ▶), EF-G (Connell *et al.*, 2007 ▶), EF-Tu (Schuette *et al.*, 2009 ▶; Villa *et al.*, 2009 ▶) as well as RF3 (Gao *et al.*, 2007 ▶) contact helix 5 of 16S rRNA in cryo-EM reconstructions of ribosomal complexes with GTPase translation factors. During the translation cycle, the two ribosomal subunits rotate by approximately 6° with respect to each other in a so-called ‘ratcheting’ movement. This GTPase–ribosome contact occurs in both ratcheted and nonratcheted conformations (Connell *et al.*, 2007 ▶; Gao *et al.*, 2007 ▶; Schuette *et al.*, 2009 ▶; Myasnikov *et al.*, 2005 ▶).

Our observations suggested to us that the preferred contact between L9 and the 30S shoulder could be one of the main causes of the lack of success in crystallizing complexes of 70S ribosomes from several different species with different ribosomal GTPases.

### 3.2. Design and construction of a *T. thermophilus* strain with a truncated ribosomal protein L9 gene

It has previously been shown that *E. coli* is viable when the chromosomal L9 gene is truncated or deleted and that the peptidyl-transferase activity of 50S subunits lacking L9 is indistinguishable from that of the wild type (Lieberman *et al.*, 2000 ▶; Herr *et al.*, 2001 ▶). The only known function of L9 is in preventing mRNA slippage (reviewed in Atkins & Björk, 2009 ▶). Ribosomes from the bacterium *T. thermophilus* have produced well diffracting crystals of 30S subunits (Wimberly *et al.*, 2000 ▶) as well as 70S ribosomes (Selmer *et al.*, 2006 ▶). Therefore, we set out to construct a *T. thermophilus* strain in which ribosomal protein L9 is truncated after the N-­terminal domain in order to eliminate its potential crystal contact with a neighbouring 30S subunit. The truncation was designed based on the L9 structure within the *T. thermophilus* 70S ribosome (Selmer *et al.*, 2006 ▶) and was intended to not perturb the N-­terminal 23S RNA-binding domain.

The *T. thermophilus* HB8-MRCMSAW1 strain was constructed using homologous recombination and has a stop codon after L9 residue 55 followed by a kanamycin-resistance cassette.

### 3.3. Crystallization of engineered *T. thermophilus* 70S ribosomes in complex with EF-G and analysis of crystal-packing interactions

Diffraction-quality crystals of the fusidic acid-locked complex of EF-G with 70S ribosomes from *T. thermophilus* strain HB8-MRCMSAW1, mRNA and P-site tRNA were grown in MES buffer pH 6.5–6.6 using PEG 20K as a precipitant (Fig. 2 ▶). There is no electron density for the N-terminal domain of L9 in these structures, indicating that the truncated version of the protein has not been incorporated into the ribosomes of the mutant strain (Gao *et al.*, 2009 ▶).

The DOBC detergent, which was added to the complex solution prior to crystallization, was critical for crystal growth in the above condition. Interestingly, the same detergent was also used in the crystallization of wild-type *T. thermophilus* 70S ribosomes with mRNA and tRNA (Selmer *et al.*, 2006 ▶), in which case detergent addition improved crystal morphology and diffraction. Using similar but not identical conditions, ribosomes lacking L9 could also be crystallized in complex with EF-Tu (Schmeing *et al.*, 2009 ▶; Voorhees *et al.*, 2010 ▶) and with RF3 (Jin *et al.*, 2011 ▶).

**Figure 4:**
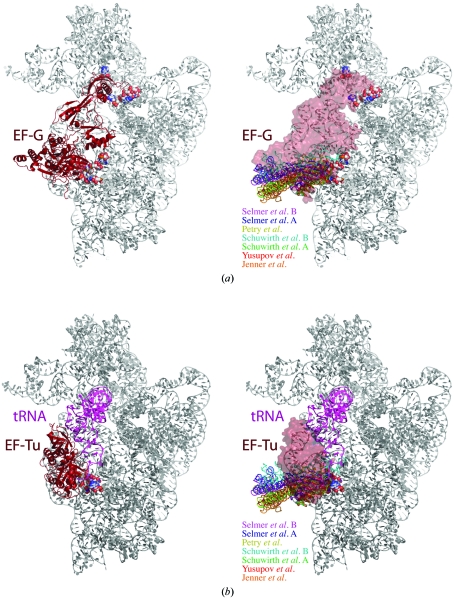
Comparison of elongation-factor interactions with the 16S rRNA shoulder and L9 crystal-packing interactions with the same region. (*a*) Fusidic acid-locked structure of EF-G in complex with the 70S ribosome (Gao *et al.*, 2009 ▶). In the left panel, EF-G is shown as a brown ribbon and the 30S ribosome is shown in grey. In the right panel, the EF-G surface is shown in transparent brown, the 30S ribosome in grey and the L9 crystal contacts in the same colours as in Fig. 3 ▶(*b*). (*b*) Kirromycin-locked structure of EF-Tu and tRNA with the 70S ribosome (Schmeing *et al.*, 2009 ▶). In the left panel, EF-Tu is shown as a brown ribbon, tRNA is shown in magenta and the 30S ribosome in grey. In the right panel, the EF-Tu surface is shown in transparent brown, the 30S ribosome in grey and the L9 crystal contacts in the same colours as in Fig. 3 ▶(*b*).

In the crystal structures of EF-G (Gao *et al.*, 2009 ▶) and EF-Tu (Schmeing *et al.*, 2009 ▶) bound to the engineered 70S ribosome, as observed in cryo-EM reconstructions, domain II of the elongation factors interacts with helix 5 and helix 15 of the 16S rRNA shoulder (Fig. 4 ▶). The exact interactions are not conserved between the fusidic acid-stalled EF-G complex and the kirromycin-stalled EF-Tu complex. The hidden surface area in the contact varies between 481 and 536 Å^2^ and the position of domain II of the two factors differs by 3–5 Å relative to 16S rRNA. For EF-Tu, this interaction has been implicated in GTPase activation in response to correct decoding (Schmeing *et al.*, 2009 ▶) and the same may be true for EF-­G (Gao *et al.*, 2009 ▶).

This work shows that there are situations in which fortuitous crystal contacts in the ribosome can be so strong that they can displace normal binding of factors and lead to crystallization without the factors present. We had previously been unable to obtain alternative crystal forms that included the factor, presumably because crystallization of the factorless forms was favoured by the L9 contact and drove the equilibrium in this direction. The fact that a deletion mutant unable to form the L9 contact could be used to crystallize three different GTPase ribosome complexes shows that engineering ribosomes to remove favourable contacts can allow the crystallization of completely new forms. It is possible that this strategy may be of use in the crystallization of other macromolecular complexes.

## Figures and Tables

**Table 1:** Crystal contacts between ribosomal protein L9 and the 30S shoulder

PDB entries and reference	Species and complex	Resolution (Å)	Hidden surface area† (Å^2^)
2j00, 2j01, 2j02, 2j03 (Selmer *et al.*, 2006 ▶)	*T. thermophilus* 70S, mRNA, 3 tRNAs	2.8	L9a–16Sb, 456; L9b–16Sa, 138
1gix, 1giy (Yusupov *et al.*, 2001 ▶)	*T. thermophilus* 70S, mRNA, 3 tRNAs	5.5	n.d.
1yl3, 1yl4 (Jenner *et al.*, 2005 ▶)	*T. thermophilus* 70S, mRNA, 2 tRNAs	5.5	462
2avy, 2aw4, 2aw7, 2awb (Schuwirth *et al.*, 2005 ▶)	*E. coli* 70S	3.5	L9a–16Sb, 199; L9b–16Sa, 438
2b64, 2b66 (Petry *et al.*, 2005 ▶)	*T. thermophilus* 70S, mRNA, 2 tRNAs, RF1	5.9	144

† L9 accessible surface area lost in interaction with 16S rRNA calculated using *AREAIMOL* in the *CCP*4 package (Winn *et al.*, 2011 ▶) for all structures for which all-atom coordinates have been deposited in the PDB.

## References

[bb1] Atkins, J. F. & Björk, G. R. (2009). *Microbiol. Mol. Biol. Rev.* **73**, 178–210. 10.1128/MMBR.00010-08 PMC2650885 19258537

[bb2] Ban, N., Nissen, P., Hansen, J., Moore, P. B. & Steitz, T. A. (2000). *Science*, **289**, 905–920. 10.1126/science.289.5481.905 10937989

[bb3] Cameron, D. M., Gregory, S. T., Thompson, J., Suh, M. J., Limbach, P. A. & Dahlberg, A. E. (2004). *J. Bacteriol.* **186**, 5819–5825. 10.1128/JB.186.17.5819-5825.2004 PMC516821 15317787

[bb5] Connell, S. R., Takemoto, C., Wilson, D. N., Wang, H., Murayama, K., Terada, T., Shirouzu, M., Rost, M., Schüler, M., Giesebrecht, J., Dabrowski, M., Mielke, T., Fucini, P., Yokoyama, S. & Spahn, C. M. (2007). *Mol. Cell*, **25**, 751–764. 10.1016/j.molcel.2007.01.027 17349960

[bb7] Gao, H., Zhou, Z., Rawat, U., Huang, C., Bouakaz, L., Wang, C., Cheng, Z., Liu, Y., Zavialov, A., Gursky, R., Sanyal, S., Ehrenberg, M., Frank, J. & Song, H. (2007). *Cell*, **129**, 929–941. 10.1016/j.cell.2007.03.050 17540173

[bb6] Gao, Y.-G., Selmer, M., Dunham, C. M., Weixlbaumer, A., Kelley, A. C. & Ramakrishnan, V. (2009). *Science*, **326**, 694–699. 10.1126/science.1179709 PMC3763468 19833919

[bb8] Hansen, J. L., Schmeing, T. M., Moore, P. B. & Steitz, T. A. (2002). *Proc. Natl Acad. Sci. USA*, **99**, 11670–11675. 10.1073/pnas.172404099 PMC129327 12185246

[bb9] Hashimoto, Y., Yano, T., Kuramitsu, S. & Kagamiyama, H. (2001). *FEBS Lett.* **506**, 231–234. 10.1016/s0014-5793(01)02926-x 11602251

[bb10] Herr, A. J., Nelson, C. C., Wills, N. M., Gesteland, R. F. & Atkins, J. F. (2001). *J. Mol. Biol.* **309**, 1029–1048. 10.1006/jmbi.2001.4717 11399077

[bb11] Hoffman, D. W., Davies, C., Gerchman, S. E., Kycia, J. H., Porter, S. J., White, S. W. & Ramakrishnan, V. (1994). *EMBO J.* **13**, 205–212. 10.1002/j.1460-2075.1994.tb06250.x PMC394794 8306963

[bb12] Hoseki, J., Yano, T., Koyama, Y., Kuramitsu, S. & Kagamiyama, H. (1999). *J. Biochem.* **126**, 951–956. 10.1093/oxfordjournals.jbchem.a022539 10544290

[bb13] Jenner, L., Romby, P., Rees, B., Schulze-Briese, C., Springer, M., Ehresmann, C., Ehresmann, B., Moras, D., Yusupova, G. & Yusupov, M. (2005). *Science*, **308**, 120–123. 10.1126/science.1105639 15802605

[bb14] Jin, H., Kelley, A. C. & Ramakrishnan, V. (2011). *Proc. Natl Acad. Sci. USA*, **108**, 15798–15803. 10.1073/pnas.1112185108 PMC3179103 21903932

[bb15] Koyama, Y., Hoshino, T., Tomizuka, N. & Furukawa, K. (1986). *J. Bacteriol.* **166**, 338–340. 10.1128/jb.166.1.338-340.1986 PMC214599 3957870

[bb16] Lieberman, K. R., Firpo, M. A., Herr, A. J., Nguyenle, T., Atkins, J. F., Gesteland, R. F. & Noller, H. F. (2000). *J. Mol. Biol.* **297**, 1129–1143. 10.1006/jmbi.2000.3621 10764578

[bb17] Myasnikov, A. G., Marzi, S., Simonetti, A., Giuliodori, A. M., Gualerzi, C. O., Yusupova, G., Yusupov, M. & Klaholz, B. P. (2005). *Nature Struct. Mol. Biol.* **12**, 1145–1149. 10.1038/nsmb1012 16284619

[bb18] Ogle, J. M., Brodersen, D. E., Clemons, W. M. Jr, Tarry, M. J., Carter, A. P. & Ramakrishnan, V. (2001). *Science*, **292**, 897–902. 10.1126/science.1060612 11340196

[bb19] Petry, S., Brodersen, D. E., Murphy, F. V., Dunham, C. M., Selmer, M., Tarry, M. J., Kelley, A. C. & Ramakrishnan, V. (2005). *Cell*, **123**, 1255–1266. 10.1016/j.cell.2005.09.039 16377566

[bb20] Schmeing, T. M., Voorhees, R. M., Kelley, A. C., Gao, Y.-G., Murphy, F. V. IV, Weir, J. R. & Ramakrishnan, V. (2009). *Science*, **326**, 688–694. 10.1126/science.1179700 PMC3763470 19833920

[bb21] Schuette, J. C., Murphy, F. V., Kelley, A. C., Weir, J. R., Giesebrecht, J., Connell, S. R., Loerke, J., Mielke, T., Zhang, W., Penczek, P. A., Ramakrishnan, V. & Spahn, C. M. (2009). *EMBO J.* **28**, 755–765. 10.1038/emboj.2009.26 PMC2666022 19229291

[bb22] Schuwirth, B. S., Borovinskaya, M. A., Hau, C. W., Zhang, W., Vila-Sanjurjo, A., Holton, J. M. & Cate, J. H. (2005). *Science*, **310**, 827–834. 10.1126/science.1117230 16272117

[bb23] Selmer, M., Dunham, C. M., Murphy, F. V., Weixlbaumer, A., Petry, S., Kelley, A. C., Weir, J. R. & Ramakrishnan, V. (2006). *Science*, **313**, 1935–1942. 10.1126/science.1131127 16959973

[bb24] Spahn, C. M., Blaha, G., Agrawal, R. K., Penczek, P., Grassucci, R. A., Trieber, C. A., Connell, S. R., Taylor, D. E., Nierhaus, K. H. & Frank, J. (2001). *Mol. Cell*, **7**, 1037–1045. 10.1016/s1097-2765(01)00238-6 11389850

[bb25] Stock, D., Perisic, O. & Löwe, J. (2005). *Prog. Biophys. Mol. Biol.* **88**, 311–327. 10.1016/j.pbiomolbio.2004.07.009 15652247

[bb26] Villa, E., Sengupta, J., Trabuco, L. G., LeBarron, J., Baxter, W. T., Shaikh, T. R., Grassucci, R. A., Nissen, P., Ehrenberg, M., Schulten, K. & Frank, J. (2009). *Proc. Natl Acad. Sci. USA*, **106**, 1063–1068. 10.1073/pnas.0811370106 PMC2613361 19122150

[bb27] Voorhees, R. M., Schmeing, T. M., Kelley, A. C. & Ramakrishnan, V. (2010). *Science*, **330**, 835–838. 10.1126/science.1194460 PMC3763471 21051640

[bb28] Wimberly, B. T., Brodersen, D. E., Clemons, W. M., Morgan-Warren, R. J., Carter, A. P., Vonrhein, C., Hartsch, T. & Ramakrishnan, V. (2000). *Nature (London)*, **407**, 327–339. 10.1038/35030006 11014182

[bb4] Winn, M. D. *et al.* (2011). *Acta Cryst.* D**67**, 235–242.

[bb29] Yusupov, M. M., Yusupova, G. Z., Baucom, A., Lieberman, K., Earnest, T. N., Cate, J. H. & Noller, H. F. (2001). *Science*, **292**, 883–896. 10.1126/science.1060089 11283358

